# Pattern of Intravenous Proton Pump Inhibitors Use in ICU and Non-ICU Setting: A Prospective Observational Study

**DOI:** 10.4103/1319-3767.70614

**Published:** 2010-10

**Authors:** Mohammed S. Alsultan, Ahmed Y. Mayet, Areej A. Malhani, Mashael K. Alshaikh

**Affiliations:** Department Chair, Clinical Pharmacy Department, College of Pharmacy, King Saud University Riyadh, Saudi Arabia; 1 Clinical Pharmacy Department, College of Pharmacy, King Saud University, Riyadh, Saudi Arabia; 2 Clinical Pharmacy Specialist King Fahad Medical City, Riyadh, Saudi Arabia; 3 Clinical Pharmacy Specialist, BCPS, King Khalid University Hospital, Riyadh, Saudi Arabia

**Keywords:** Intensive care, acid-suppressive, therapy, prophylaxis, stress ulcer

## Abstract

**Background/Aim:**

The use of intravenous acid-suppressive therapy for stress ulcer prophylaxis in critically ill patients with specific risk factors has been recommended for over a decade. However, there is a lack of supporting data regarding the extension of such therapy to non-critically ill patients (non-ICU). The aim of this study was to compare appropriate indications with current practicing patterns in adult non-ICU and ICU patients, contributing factors and financial impact of inappropriate use.

**Materials and Methods:**

A prospective cross-sectional study was carried out at a tertiary teaching Hospital in Riyadh, Saudi Arabia. For a period of 4 consecutive months, all hospitalized patients on IV PPI, aged 18 and above, were identified. A concise listing of indications considered appropriate for the use of IV PPI was pre-defined based on material from available literature and guidelines.

**Results:**

A total of 255 patients received IV PPI. Inappropriate use of IV PPI was significantly higher in non-ICU (71.7%) than in ICU (19.8%) patients (*P*=0.01). The most common cause for inappropriate use in non-ICU patients was stress ulcer prophylaxis (SUP). In ICU patients, appropriate indicators for IV PPI were SUP (47.9%), PUD (11.5%), and the UGIB (20.8%). There was a high association between appropriate uses of IV PPI with respect to endoscopic procedure and also between appropriate uses of IV PPI to subsequent discharge with oral PPI in non ICU patients. The total estimated direct cost (drug acquisition cost) for inappropriate use of IV PPI during the study period was 11,000 US dollars.

**Conclusion:**

Inappropriate IV PPI utilization was predominant in non-ICU patients, mostly for stress ulcer prophylaxis that leads to a waste of resources. Applying appropriate policies, procedures and evidence-based guidelines, educated physicians and surgeons can clearly limit inappropriate IV PPI use.

Proton pump inhibitors (PPIs) are one of the most commonly prescribed classes of medications. Their superior acid suppression provides better control of intra-gastric pH over a 24-h period, compared with histamine type-2 receptor antagonists (H2RA).[1] Omeprazole was the first PPI introduced to the market, quickly followed by lansoprazole, rabeprazole, pantoprazole, and esomeprazole. These products are used widely in the management of acid-related disorders, and in the majority of patients, oral therapy proves highly effective. At equivalent doses oral and intravenous (IV) PPIs produce comparable acid suppression.[2]

There are very few clinical indications for IV PPI therapy. It is indicated in exceptional circumstance as follows: in patients with gastric hypersecretion associated with neoplastic conditions and Zollinger-Ellison syndrome unable to take oral medication; in severe non-variceal upper gastrointestinal bleeding; gastrointestinal bleeding at risk for continuous recurrent bleeding, and in prevention of stress-related mucosal disease bleeding (stress ulcer prophylaxis) in high risk ICU patients without enteral feeding access or “nothing by mouth” status.[3–7]

The practice of using IV PPI as a stress ulcer prophylaxis (SUP) in the ICU setting has been extrapolated to the care of non-ICU patients, without there being any evidence to support this need.[3,7] In 2006, Heidelbaugh *et al*. conducted a retrospective charts review, aiming to examine the practice of SUP in non-ICU patients in the United States. The study was carried out on adult non-ICU admissions at one family medicine and five general internal medicine teaching services over a consecutive 4-month period. The study showed that of the 1,769 patient admissions, 22% received SUP, even though none of these patients met evidence-based criteria for appropriate SUP. Such SUP over utilization cost the hospital $44,096.[8] Also, several other recent retrospective studies showed that SUP is over-utilized in non-ICU settings and patients are often prescribed anti-secretory therapy (AST) unnecessarily. Patients are then often discharged from hospital on these medications, resulting in a significant increase in cost expenditure and exposure to risk of adverse effects including pneumonia and hip fracture.[9–14]

American Society of Health System (ASHP) guidelines for stress ulcer prophylaxis (SUP) from 1999 serve as a framework for instituting preventive therapy in ICU patients.[3] Stress ulcer prophylaxis is not recommended for adult general medical and surgical patients in non-ICU settings with fewer than two risk factors for clinically important bleeding, including severe trauma with spinal cord injury, overt sepsis, or history of gastric ulceration or bleeding during the year prior to admission or for patients with two or more risk factors.[8,15,16] An evidence-based study has demonstrated that 900 ICU patients need to be treated to prevent a single episode of clinically significant bleeding.[17] In critically ill patients, approximately 75% of patients have endoscopic evidence of stress-related mucosal disease within 24 h of ICU admission, and lesions usually resolve unaided as the condition of the patient stabilizes. However, stress-related mucosal bleeding occurs, factors including length of hospitalization, cost and mortality rate increase.[4,18]

Few studies to date have effectively examined the role of SUP in non-ICU patients, and cost-effectiveness studies in patients receiving SUP have been limited solely to ICU settings. Thus, this study was carried out with the following objectives.

- For intravenous proton pump inhibitors, investigate appropriate (evidence-based) indications to current prescribing patterns in adult non-ICU and in ICU patients.
- To observe the characteristics of patients, hospital stay, place of prescribing (department), and speciality.
- Association between appropriate and inappropriate use of IV PPI and endoscopic procedure.
- Association between appropriate and inappropriate use of IV PPI and subsequent discharge with oral PPI in non-ICU patients.

Assess contributing factors and financial impact of inappropriate use. A concise listing of indications considered appropriate (evidence-based) for use of IV PPI were pre-defined from the available literature and guidelines.

## MATERIALS AND METHODS

This was a prospective cross-section observational study conducted at a tertiary teaching hospital in Riyadh, Saudi Arabia. This 500+ bed tertiary teaching medical center has an average census of 400 non-intensive care [hospitalized adult medicine (Non-ICU) patients], and 100+ patients in surgical, medical, and cardiac intensive care units (ICU). The patient population is predominantly local citizens, but the hospital serves the entire country as a referral centre. Medical care is supervised by attending physicians and delivered by medical residents, interns, and students.

During a 4-month period, (August to November 2008) all hospitalized patients on IV PPI’s at our medical centre were identified on a daily basis from pharmacy records. Patients aged 18 and above in the 14 non-ICU wards and 6 ICU wards were included in the study. Medical records (files) of study subjects were reviewed and the following information was collected for analysis: demographic data (age, gender); diagnosis; drugs history; previous peptic ulcer history; admission and discharge date; PPI regimen and clinical indication; upper gastrointestinal endoscope findings; nothing by mouth and mechanical ventilation status; prescriber’s department and specialty; concomitant, and discharge medications. Indications considered appropriate (evidence-based) for use of IV PPI were pre-defined. Appropriate (evidence-based) indication for IV PPI.[3,7,8]

1. Prevention of stress-related mucosal disease bleeding in critically ill and or mechanically ventilated patients (ASHP guideline for SUP).
2. Treatment of active upper gastrointestinal bleeding (UGIB).
3. Treatment of peptic ulcer diseases (PUD).

### Statistical analysis

The collected data were coded and entered into the Statistical Package for the Social Science (SPSS version 11) for analysis. The patients’ age, length of PPI use, departments, and specialties were formulated into category variables. The chi-square and Fisher’s exact tests were used to compare appropriate and inappropriate use of IV PPI with respect to age, gender, duration of use, department, endoscope data, and discharge medications in non-ICU patients. We have also used the Z-test of percents drawn from one sample to compare the percentage of difference in appropriate and inappropriate use of IV PPI in ICU, non-ICU, and specialty

## RESULTS

A total of 255 patients who received IV PPI were identified during the study period and their files were reviewed. The mean age of the patients in the study was 42 ± 21 years, and 121 (47.5%) of the patients were males. Of these, 159 patients were from non-ICU and 96 patients were from ICU.

In non-ICU setting, we found a significantly higher number of patients, 114 (71.7%) received IV PPI inappropriately as SUP without meeting the SUP criteria, compared to 45 (28.3%) who received it appropriately (*P*=0.01) for PUD and UGIB. No significant difference was found between appropriate and inappropriate use of IV PPI in non-ICU male and female patients (*P* = 0.29) [Tables 1 and 2].

**Table 1:** Indication for use of IV PPI (appropriate and inappropriate) in ICU and non-ICU

Study variable	Appropriate	Inappropriate	*P* value
Non-ICU (N = 159)	45 (28.3)	114 (71.7)	0.01
*SUP*	-	114 (71.7)	
*UGIB*	29 (18.2)	-	
*PUD*	16 (10.1)	-	
ICU (N=96)	77 (80.2)	19 (19.8)	0.01
*SUP*	46 (47.9)	19 (19.8)	
*UGIB*	20 (20.8)	-	
*PUD*	11 (11.5)	-	
Total (N=255)	122 (47.8)	133 (52.2)	

Figures in parenthesis are in percentage

**Table 2:** Association between appropriate and inappropriate use of IV PPI in non-ICU and study variables

Study variables	Appropriate N=45 (28.3)	Inappropriate N=114 (71.6)	Total 159 (100%)	χ^2^ -value	*P* value
Age group					
<40	18 (40.0)	45 (39.4)	63 (39.7)	0.37	0.95
>40	27 (60.0)	69 (60.6)	96 (60.3)		
Gender					
Male	25 (55.6)	51 (44.8)	76 (47.8)	1.51	0.291
Female	20 (44.4)	63 (55.2)	83 (52.2)		
Duration of use(days)					
<8	40 (88.9)	87 (76.3)	127 (79.9)	3.17	0.075
>8	5 (11.1)	27 (23.7)	32 (20.1)		
Specialty*					
Consultant	19 (26.4)	53 (73.6)	72 (45.3)		0.0001
Registrar	14 (30.4)	32 (69.6)	46 (28.9)		0.006
Specialist	7 (25.5)	20 (74.1)	27 (17.0)		0.0085
Resident	5 (35.7)	9 (64.3)	14 (8.8)		0.286

* Applied Z test*

We also found no significant difference for non-ICU patients in the following groups: between two different age groups (less than or equal to 40 and more than 40 years of age) in relation to appropriate and inappropriate use of IV PPI *P*=0.95; duration of appropriate and inappropriate IV PPI use between less than or more than 8 days *P*=0.118; and appropriate and inappropriate prescribing of IV PPI amongst different departments (surgery, cardiology, medicine, surgery, oncology) *P*=0.082, respectively [Tables 2 and 3].

**Table 3:** Association between appropriate and inappropriate use of IV PPI in non-ICU and various departments

Reason	Department					Total n = 159	χ^2^ value	*P* value
	Cardiology	Medicine	Neurology	Oncology	Surgery			
Appropriate UGIB + PUD	4	9	2	17	13	45	8.27	0.082
Inappropriate SUP	27	33	4	31	19	114		

We observed a highly significant association between appropriate use of IV PPI with respect to endoscopic procedure (*P* = 0.0001). Forty two (93.3%) patients who received IV PPI appropriately in non-ICU underwent an upper gastrointestinal endoscope procedure, whereas 103 (90.4%) patients who received IV PPI inappropriately did not. Lastly, we observed a highly significant association between appropriate use of IV PPI and subsequent discharge with oral PPI in non-ICU patients (*P*=0.0001). Forty-three (95.6%) from appropriate IV PPI recipients compared with 50 (43.9%) inappropriate IV PPI recipients were discharged with appropriate continuous oral PPI.

A Z-test of percents drawn from sample was used for analysis and allowed us to conclude that there was a significant difference in the prescribing of appropriate and inappropriate IV PPI in non-ICU patients by the person attending of different specialties (consultant, registrar, and specialist) (*P* = 0.006) [Table 2].

In ICU patients, a significantly higher number of patients, 77 (80.2%), received IV PPI appropriately, compared to 19 patients (19.8%) inappropriately (*P* = 0.01). Among appropriate IV PPI recipients, 20 (20.8%) had endoscopically proven UGIB, 11 (11.5%) had PUD, and 46 (47.9%) were on a mechanical ventilator with nothing by mouth (NPO) status who required SUP. Only 19 (19.8%) ICU patients received IV PPI inappropriately as SUP without indication [Table 1].

Upon discharge, 21 (22%) ICU patients who received IV PPI for SUP and 7 patients (8%) who received IV PPI inappropriately were unnecessarily switched to oral PPI and released.

The total direct cost (drug acquisition cost) for inappropriate use of IV PPI during the study period for inpatients was 11,000 US dollars. Assuming a similar prescribing pattern will be continued, the extrapolated cost per year will reach an approximate of 44,000 US dollars. The cost of inappropriate use of IV PPI from non-ICU was significantly higher than of ICU.

## DISCUSSION

ASHP guidelines for SUP serve as a framework for instituting preventive therapy in ICU patients.[3,7] The guidelines do not recommend routines involving antisecretory therapy (IV H2RA or IV PPIs) for stress ulcer prophylaxis, except in critically ill patients (ICU setting) with specific risk factors, yet this practice has been extended to non-ICU patient populations for SUP without supportive data, thereby burdening hospitals with excessive cost.[10–19] Our 4-month study highlights the common practice of inappropriate IV PPI use in non-ICU patients and ICU patients at our medical center despite a lack of evidence supporting its use, and it is notable that most of the inappropriate use (71.7%) occurred in a non-ICU setting similar to the situation reported by others. In 2003, Schupp *et al*. studied 814 general adult medicine patients in a community hospital setting; 324 were given IV PPI for stress ulcer prophylaxis. The authors noted that 40% of the patients were actually given IV PPI for an appropriate indication, while 60% were not. The most frequently cited reason for prescribing PPI without an indication was “GI prophylaxis” in his study, while the main reason for inappropriate use in our study was also as SUP without meeting the criteria.[20] It is our impression that the reason for prescribing IV PPI in the non-ICU setting for SUP is that medical doctors consider certain non-ICU patients to be at a higher risk of developing stress ulcers specially those patients who are on nonsteroidal anti-inflammatory drugs, aspirin, corticosteroids, receiving chemotherapy and elderly. Therefore, a fairly simple intervention of using IV PPI may protect against stress ulceration, but there are no data supporting the benefit of using it. This practice resulted in an estimated direct cost of 44,000 US dollars annually for inappropriate usage of IV PPI at our institute, representing a substantial cost expenditure that could have been avoided. The cost calculation did not include intravenous solution, tubing, hospital stay, pharmacy preparation, and nursing cost. Erstad *et al*. reported similar findings in a 3-month study period evaluating 693 ICU patients who were treated for SUP; 607 (87.6%) received IV PPI, and 86 (12.4%) H2RA. Seventy-two patients (10%) were on a mechanical ventilator and 48 (6.9%) had coagulopathy, while the remaining 573 (82.7%) did not have either risk factor. Inappropriate use of acid-suppressing agents during the study period was $15,760 with the 1-year extrapolated cost being $63,000.[21]

In addition to no adequate indication for IV PPI use in ICU and non-ICU patients, these patients were also discharged on oral PPI without reason. Strid *et al*., in a comparative study, demonstrated 41% (120) patients with chronic renal failure on dialysis who were on IV PPI for SUP, 63% of the cases, there was no adequate indication for IV PPI use. Thirty-four percent (34%) of patients in the study who received IV PPI for SUP were discharged on oral PPI.[22] In our study, 78 (30.1%) (50 non-ICU and 28 ICU) patients were also discharged on oral PPI without reason. If we estimated annual cost expenditures based on an assumption that a constant proportion of our patients receive IV PPI as inpatients and discharged on oral PPI therapy (30.1%), the cost was around $8,000, based on a 1 month supply. The cost calculation (drug acquisition cost) was limited to the prescriptions written for the patients during the time of discharge and did not include refills and dispensing charges.

Our non-ICU data also demonstrated that of patients who underwent the upper gastrointestinal endoscope procedure, 95% received oral PPI appropriately during discharge. Therefore, performing an upper gastrointestinal endoscope in such patients may guide the physicians to long-term use of oral antisecretory agents (AST).

There were a large number of prescriptions written by cardiologists and oncologists in our non-ICU patients for SUP. The most likely reason for this was that their patients were either on aspirin for ischemic heart disease or anticoagulant for thromboembolism prophylaxis or developed blood dyscrasia from chemotherapy and so were at a higher risk of developing SUP. Unlike our study, Strid *et al* in their study, showed that inappropriate prescriptions for acid suppressive therapy were largely written by nephrologists, followed by rheumatologists and pulmanologists.[22]

We noted a significant difference between appropriate and inappropriate prescribing of IV PPI among different specialties (consultant, registrar, and specialist) except by resident in our study but no difference was found in appropriate and inappropriate prescribing among different departments (surgery, cardiology, oncology, medicine, or surgery). We were unable to explain the discrepancy in prescribing habits between different specialties.

Resource utilization data are an essential component of the cost effect utilization of medication in an institution. The data regarding stress ulcer prophylaxis trends in the ICU setting have been published. A study performed at the Carolinas Medical Centre found an estimated annual saving of $102,895 in patient charges and $11,333 in actual drug costs, in a trauma ICU attributable to the implementation of stress ulcer prophylaxis guidelines.[23] We could not find any resource utilization data in a non-ICU setting. In the future, research should focus on the resource utilization in non-ICU for stress ulcer prophylaxis (SUP) which may shed light on the magnitude of the problem and on cost saving.

## CONCLUSION

In our study, a significant number of non-ICU patients received IV PPI inappropriately for SUP, indicating that our hospital, like others, experienced widespread misuse of IV PPIs in hospital practices, leading to a waste of resources.

Therefore, we suggest that individual hospitals should develop their own potential intervention strategies to minimize inappropriate use of IV PPI including utilization of ASHP guidelines for SUP in non-ICU patients and developing policy and procedures to restrict its use with the participation of gastroenterologists, critical care physicians, and clinical pharmacists without compromising patient care. Also hospitals should consider developing controlled policies like formulary restriction, stop-orders for specific indications, and automatic switch-order to oral PPI if patient is receiving oral feeding. Educating physicians and surgeons through newsletter and electronic email alert detailing appropriate indications (evidenced-base) of IV PPI can also reduce the misuses of IV PPI.
